# The RET inhibitor pralsetinib suppresses TMZ-resistant glioma growth by regulating spermine production

**DOI:** 10.3389/fphar.2025.1671798

**Published:** 2025-10-31

**Authors:** Lingyun Ma, Hang Gong, Wei Sun, Jialing Deng, Qinghua Zhang, Jianzhao Niu, Huimin Sun, Xue Han, Tingting Du, Nina Xue, Ming Ji, Qian Liu

**Affiliations:** ^1^ NMPA Key Laboratory for Quality Research and Evaluation of Chemical Drugs, National Institutes for Food and Drug Control, Beijing, China; ^2^ Department of Pharmacology, Institute of Materia Medica, Chinese Academy of Medical Sciences and Peking Union Medical College, Beijing, China; ^3^ Department of Neurology, Ningguo City People’s Hospital, Ningguo, China; ^4^ National Engineering Research Center for the Development of New Drugs, Institute of Materia Medica, Chinese Academy of Medical Sciences and Peking Union Medical College, Beijing, China

**Keywords:** Glioma, TMZ resistance, Spermine, Spermine Synthase, Pralsetinib

## Abstract

**Objective:**

Glioma is the most common malignant tumor of the central nervous system and is characterized by altered cellular metabolism. Although temozolomide (TMZ)-based adjuvant treatment has improved overall patient survival, clinical outcomes remain unsatisfactory. TMZ resistance is a major contributing factor. The mechanisms underlying TMZ resistance are highly complex. This study aimed to elucidate the role of spermine in TMZ resistance and to assess the antitumor activity of kinase inhibitors against TMZ-resistant glioma.

**Methods:**

The TCGA data from glioma patients treated with TMZ was analyzed and metabolomic analysis of both TMZ-sensitive and TMZ-resistant glioma cells was performed. A series of compounds in both TMZ-sensitive and TMZ-resistant glioma cells were screened, and their brain penetration capacity was tested by MacroFlux assay. The antitumor activity of pralsetinib was evaluated *in vitro* and *in vivo*.

**Results:**

In this study, we found that spermine synthase (SMS) was highly expressed in gliomas that showed a poor clinical response to TMZ treatment. Spermine, the metabolic product of SMS, was also elevated in TMZ-resistant glioma cells and promoted their proliferation. Further investigation revealed that pralsetinib, a selective RET inhibitor, exhibited significant antitumor activity against TMZ-resistant glioma cells both *in vitro* and *in vivo*. Mechanistically, pralsetinib inhibited the spermine-induced activation of the PI3K/AKT pathway and downregulated SMS expression, leading to reduced spermine production.

**Conclusion:**

Our findings reveal the role of spermine in TMZ-resistant glioma and suggest a potential new pharmacological application for pralsetinib in the glioma treatment.

## 1. Introduction

Metabolic reprogramming is a major hallmark of cancer. Glioma, the most common intracranial malignancy in adults, accounts for 31.1% of all primary brain tumors in China (Jiang et al., 2011), and relies mainly on glucose glutamine/glutamate, and fatty acids for fuel (Shen et al., 2015; Dong and Cui, 2019; Bernhard et al., 2023; Kou et al., 2022; Quinones and Le, 2018; Su et al., 2018). Polyamines, which include putrescine, spermidine, and spermine, are polycationic alkylamines commonly found in all living cells. They play important roles in many biological processes, including cell growth, proliferation, migration, gene regulation, and the synthesis of proteins and nucleic acids. Polyamine metabolism is dysregulated in brain tumors and drives carcinogenesis (Casero et al., 2018; Khan et al., 2021; Miska et al., 2021; Moulinoux et al., 1984). The first key enzyme in polyamine biosynthesis is ornithine decarboxylase (ODC), which catalyzes the conversion of ornithine to putrescine. Spermidine and spermine are then produced by the enzymatic activities of spermidine synthase (SRM) and spermine synthase (SMS), respectively. Targeting polyamine metabolism has long been an attractive strategy for anticancer therapy. For example, DFMO, a small-molecule inhibitor of ODC, was initially approved for the treatment of *Trypanosoma brucei gambiense* infection and was recently approved for patients with neuroblastoma (Levin et al., 2018; Saulnier Sholler et al., 2015; Bassiri et al., 2015; Tangella et al., 2023).

Aberrant activation of numerous signaling transduction pathways, including the overexpression or mutation of receptor tyrosine kinases (RTKs), drives glioma cell growth, survival, migration, invasion, and angiogenesis (Tilak et al., 2021). These oncogenic signaling pathways also regulate tumor metabolism in malignant glioma. The EGFR/PI3K/AKT pathway is involved in lipid metabolism in glioma and promotes tumor growth (Guo et al., 2010; Guo et al., 2013). PI3K signaling can regulate polyamine production (Rajeeve et al., 2013). Conversely, and importantly, high levels of polyamines can activate oncogenic PI3K/AKT signaling (Dai et al., 2017).

Pralsetinib and selpercatinib, which are highly selective rearranged during transfection (RET) inhibitors, have been approved by the FDA for the treatment of metastatic RET fusion-positive non-small cell lung cancer, advanced RET-mutant medullary thyroid cancer, and advanced RET fusion-positive thyroid cancer (Kim et al., 2021; Duke et al., 2023; Bradford et al., 2021; Wright, 2020). RET is involved in the physiological development of several organ systems. The activation of RET via gene fusions or point mutations is a potent oncogenic driver in these cancers and in others, such as endometrial and breast cancer (Adashek et al., 2021). Aberrant activation of RET leads to the activation of multiple downstream pathways, including RAS/MAPK, PI3K/AKT, PKA, and PKC, driving cancer development. Furthermore, high RET expression has been observed in glioma (Wiesenhofer et al., 2000), and RET fusions, including CCDC6-RET, have also been detected (Woo et al., 2020). Notably, a patient with an isocitrate dehydrogenase wild-type glioma harboring a RET amplification achieved a near-complete response to the RET inhibitor selpercatinib (Czech et al., 2022).

In this study, we found that the expression of SMS and the levels of its product spermine were elevated in TMZ-resistant or unresponsive gliomas compared to their TMZ-sensitive counterparts. The RET inhibitor pralsetinib exhibited potent antitumor activity against TMZ-resistant glioma *in vitro* and *in vivo*, regardless of RET expression levels. Interestingly, pralsetinib inhibited spermine-induced phosphorylation of AKT in TMZ-resistant glioma cells. Furthermore, pralsetinib downregulated SMS expression at both the mRNA and protein levels in T98G cells. Consistent with this, spermine levels were also reduced *in vivo* following pralsetinib treatment.

## 2 Materials and methods

### 2.1 Reagents

Pralsetinib (for *in vitro* studies) was purchased from TargetMol (Wellesley Hills, MA, USA), while pralsetinib capsules (for *in vivo* studies) were obtained from Blueprint Medicines Corporation (Cambridge, MA, USA). Other compounds, including a panel of tyrosine kinase inhibitors (anlotinib, afatinib, apatinib, axitinib, cabozantinib, ceritinib, erlotinib, gefitinib, nilotinib, osimertinib, sunitinib, and zanubrutinib) and fluphenazine, were sourced from the National Institutes for Food and Drug Control, China. The CellTiter-Glo Luminescent Cell Viability Assay kit was purchased from Promega Corporation (Madison, WI, USA). High-glucose Dulbecco’s modified Eagle’s medium (DMEM) and fetal bovine serum were obtained from Corning Life Sciences (Glendale, AZ, USA) and GeminiBio (West Sacramento, CA, USA), respectively. Primary antibodies against AKT, phospho-AKT (Ser473), ERK1/2, phospho-ERK1/2 (Thr202/Tyr204), S6RP, phospho-S6RP (Ser235/236), RET, MGMT, and β-actin were sourced from Cell Signaling Technology (Danvers, MA, USA). The antibody against spermine synthase (SMS) was obtained from Proteintech (Rosemont, IL, USA).

### 2.2 Cell lines and cell culture

The U87MG, U251, and A172 glioma cell lines were purchased from the China Infrastructure of Cell Line Resources (Beijing, China). The U118, U138, and T98G cell lines were obtained from the American Type Culture Collection (ATCC, Manassas, VA, USA). The U343, U373, and TMZ-resistant U251 (U251/T) cells were maintained in our laboratory. The U87MG, U251, and U251/T cells were cultured in Minimum Essential Medium (MEM) supplemented with 1% nonessential amino acids (NEAA). All other cell lines were cultured in DMEM. All media were supplemented with 10% FBS, 100 U/mL penicillin, and 100 µg/mL streptomycin. All cells were maintained at 37 °C in a humidified atmosphere containing 5% CO_2_. All cell lines were routinely tested and confirmed to be free of *mycoplasma* contamination using a detection kit (InvivoGen, Hong Kong, China).

### 2.3 Cell viability assay

Cell viability was assessed using the CellTiter-Glo Luminescent Cell Viability Assay. Briefly, cells were plated in 96-well culture plates at a density of 3 × 10^3^ cells per well and incubated overnight to allow for adhesion. The cells were then treated with a range of pralsetinib concentrations, along with a vehicle control (DMSO), in triplicate. After 72 h of incubation, 30 μL of CellTiter-Glo reagent (Promega, Beijing, China) was added to each well. The plate was mixed on an orbital shaker for 2 min to induce cell lysis, and subsequently incubated at room temperature for 10 min. Luminescence was recorded using a BioTek Synergy H1 plate reader. Half-maximal inhibitory concentration (IC_50_) values were calculated using GraphPad Prism software (version 8.1, GraphPad Software, San Diego, CA, USA).

### 2.4 RNA-seq data acquisition

RNA-seq expression data (TCGA-LGG) along with the corresponding clinical information for glioma that received TMZ treatment were programmatically downloaded from The Cancer Genome Atlas (TCGA) database using the TR-DB tool (http://ctrdb.cloudna.cn/home). Differential gene expression analysis of *ODC*, *SRM*, *SMS,* and *MGMT* among different clinical response groups was performed using a T-test.

### 2.5 Quantitative real-time PCR

Total RNA was extracted from pralsetinib-treated U87MG and T98G cells using an RNA rapid extraction kit (Yeasen Biotech, China) according to the manufacturer’s instructions. The concentration and purity of the RNA were measured. In total, 2 μg of the purified RN were reverse-transcribed into cDNA using Perfect Start Green qPCR SuperMix. Quantitative real-time PCR (qRT-PCR) was then performed using the Hieff qPCR SYBR Green Master Mix Kit (Yeasen Biotech) on an Analytik Jena Real-Time PCR System. The reaction was carried out in a volume of 20 μL, containing 10 μL of Master Mix, 0.4 μM of each primer, and 2 μL of the cDNA template. The primer sequences were as follows: *SMS* forward 5′-TGG​GCG​GGT​GAA​ACG​ATT​AC-3′, reverse 5′- CCA​AAC​TGC​TTC​GAG​TGT​AGA​A-3’. The PCR cycling protocol was as follows: initial denaturation at 95 °C for 5 min; followed by 40 cycles of 95 °C for 10 s, 55 °C for 20 s, and 72 °C for 20 s. A melt curve analysis was performed to confirm the specificity of amplification. All experiments were performed in triplicate. Threshold cycle (Ct) values for the target gene (*SMS)* and the internal reference gene (*18S rRNA*) were determined, and the relative gene expression was calculated using the 2^^(−ΔΔCt)^ method.

### 2.6 Immunoblotting analysis

Lysates from cells or tumor tissues were prepared using RIPA lysis buffer supplemented with protease and phosphatase inhibitors. The protein concentration was determined using a BCA assay. Equal amounts of protein were separated by sodium dodecyl sulfate-polyacrylamide gel electrophoresis (SDS-PAGE) and then transferred to nitrocellulose membranes. The membranes were blocked with 5% (w/v) nonfat dry milk in Tris-buffered saline with 0.1% Tween 20 (TBST) for 1 h at room temperature. Subsequently, the membranes were incubated with the indicated primary antibodies diluted in blocking buffer overnight at 4 °C. The following day, the membranes were washed three times with TBST and then incubated with the appropriate horseradish peroxidase (HRP)-conjugated secondary antibodies for 1 h at room temperature. After incubation, the membranes were thoroughly washed with TBST again. The protein bands were visualized using a Tanon ECL Western blotting Substrate kit according to the manufacturer’s instructions and detected using a chemiluminescence imaging system (Tanon 5200Multi, Tanon Life Science, Shanghai, China).

### 2.7 Apoptosis analysis

Cells were seeded into 6-well plates at a density of 2 × 10^5^ cells per well and allowed to adhere overnight. The next day, the cells were treated with 1, 2.5, or 5 μM pralsetinib for 24 h. To analyze apoptosis, both the culture medium (containing any detached cells) and the trypsinized adherent cells were collected, combined, and washed with PBS. The cell pellets were resuspended in 100 μL of 1× binding buffer. Subsequently, 5 μL of Annexin V-FITC was added, and the cells were incubated for 30 min at room temperature in the dark. Then, 5 μL of propidium iodide (PI) was added, followed by a 5-min incubation. Apoptosis was assessed immediately using a BD FACSVerse flow cytometer (BD Biosciences), and the data were analyzed with FlowJo software.

### 2.8 Animal study

Female BALB/c athymic nude mice, aged 8–10 weeks (SPF Biotech, Beijing, China), were subcutaneously implanted with 1 × 10^7^ U343 or T98G cells suspended in 0.2 mL of Matrigel solution in their right flanks. When the tumor volume reached approximately 1,000 mm^3^, the tumor tissues were harvested aseptically, and the tumor cells were isolated from the tissue homogenate. Subsequently, each mouse was implanted with 2 × 10^6^ tumor cells in the right flank. Six days later, when the average tumor volume reached approximately 100 mm^3^, the mice were randomized into groups, and treatment was initiated. Tumor-bearing mice received either vehicle, TMZ, or pralsetinib (delivered via capsules in water). TMZ was administered orally for 5 consecutive days at the dose of 50 mg/kg, while pralsetinib was administered orally at the doses of 15, 30, and 60 mg/kg daily. Tumor volume and body weight were measured twice weekly. Tumor volume was calculated using the formula: **V = 1/2 × L × W** ^**2**^, where **L** represents the longest diameter and **W** is the shortest diameter perpendicular to **L**. Data, including tumor volume and body weight, were collected and analyzed using GraphPad Prism 8.

All procedures were approved by the Ethics Committee for Animal Experiments of the Institute of Materia Medica, Chinese Academy of Medical Sciences, and Peking Union Medical College, and were conducted under the Guidelines for Animal Experiments of Peking Union Medical College (Beijing, China). The approval number is 00008957.

### 2.9 MacroFlux assay for evaluating the brain permeation of compounds

Instruments, including µFlux drug permeability apparatus (Pion Inc., Billerica, MA, USA); Mettler XPEX206 1/100,000 electronic scales, and Mettler Toledo pH meter (Mettler Toledo, Greifensee, Switzerland) were used. The blood–brain barrier (BBB) lipid solution and brain sink buffer were obtained from Pion Inc. (Billerica, MA, USA). A schematic diagram of the µFlux apparatus is shown in Supplementary Figure S1. Briefly, the test compound was diluted in DMSO at a concentration of 3.0 mg/mL as a compound stock solution, and 16 mL of phosphate buffer solution (PBS) and brain sink buffer (BSB) were accurately added to the donor chamber and the acceptor chamber, respectively. The two chambers were separated by a 1.65-cm^2^ membrane impregnated with 20 μL of BBB lipid solution to form a lipophilic barrier between the donor and acceptor chambers. The compound stock solution was diluted with PBS or BSB at five concentrations, and tested at a wavelength range of 200–720 nm using the µFlux drug permeability apparatus according to the ultraviolet spectrophotometry method. Standard curves were calculated. Then, the compound stock solution was added to the donor chamber at a stirring speed of 250 rpm at 37 °C. The drug concentration in the donor and acceptor chambers was measured by using the µFlux drug permeability apparatus at a wavelength range of 200–720 nm, and the BBB permeability of the compounds was calculated according to the equation Pe = dc/dt × V ÷ (A × C_t_ × 60), where Pe is the permeability of the drug (cm/s), dc/dt is the slope of the concentration–time profile in the acceptor chamber (µg·mL/min); V is the volume of the acceptor chamber (mL); A is the membrane area (cm^2^), and Ct is the average drug concentration in the donor chamber (µg/mL). A compound was considered to have good brain penetration when its Pe value was greater than 1 × 10^−4^ cm/s.

### 2.10 Detection of polyamine content in tumor tissues

Stock solutions of putrescine (PUT), spermidine (SPD), and spermine (SPM) were prepared in water at a concentration of 10 mg/mL. Subsequent working solutions at various concentrations were diluted from these stock solutions using a 50% methanol–water solution. For the construction of standard curves, 5 µL of each working solution was added to 95 µL of the blank surrogate matrix. The surrogate matrix used was 5% BSA in PBS for SPD and SPM and normal saline for PUT. Then, 500 µL of acetonitrile containing 0.5% formic acid and the internal standard (labetalol, 200 ng/mL) was added to each standard sample. The mixture was then vortexed and centrifuged at 14,000 rpm for 5 min. Finally, 1 µL of the supernatant was injected for LC-MS/MS analysis.

Tumor tissues were homogenized in ice-cold normal saline at a ratio of 1:5 (g:mL) to produce tissue homogenates. A 40-µL aliquot of the homogenate was then mixed with 200 µL of acetonitrile containing 0.5% formic acid and the internal standard (labetalol, 200 ng/mL). The mixture was vortexed vigorously and centrifuged at 14,000 rpm for 5 min. Subsequently, 1 µL of the final supernatant was subjected to LC-MS/MS analysis.

Quantitative analysis of the compounds was performed using an LC-MS/MS system. Chromatographic separation was achieved on a Waters ACQUITY UPLC HSS T3 column (50 mm × 2.1 mm, 1.8 µm) maintained at 40 °C. The mobile phase consisted of water with 0.5% formic acid and methanol with 0.1% formic acid, running at a flow rate of 0.3 mL/min. Detection was carried out in the positive ion mode using multiple reaction monitoring (MRM).

### 2.11 Statistical analysis

GraphPad Prism 8 software was used for data analysis. The P value was calculated using a Student’s unpaired t-test, and the IC_50_ value was calculated using nonlinear regression analysis. For all statistical analyses, ^*^p < 0.05, ^**^p < 0.01, and ^***^p < 0.001 were considered to indicate statistical significance.

## 3 Results

### 3.1 Spermine expression was upregulated in TMZ-resistant glioma cells

**FIGURE 1:**
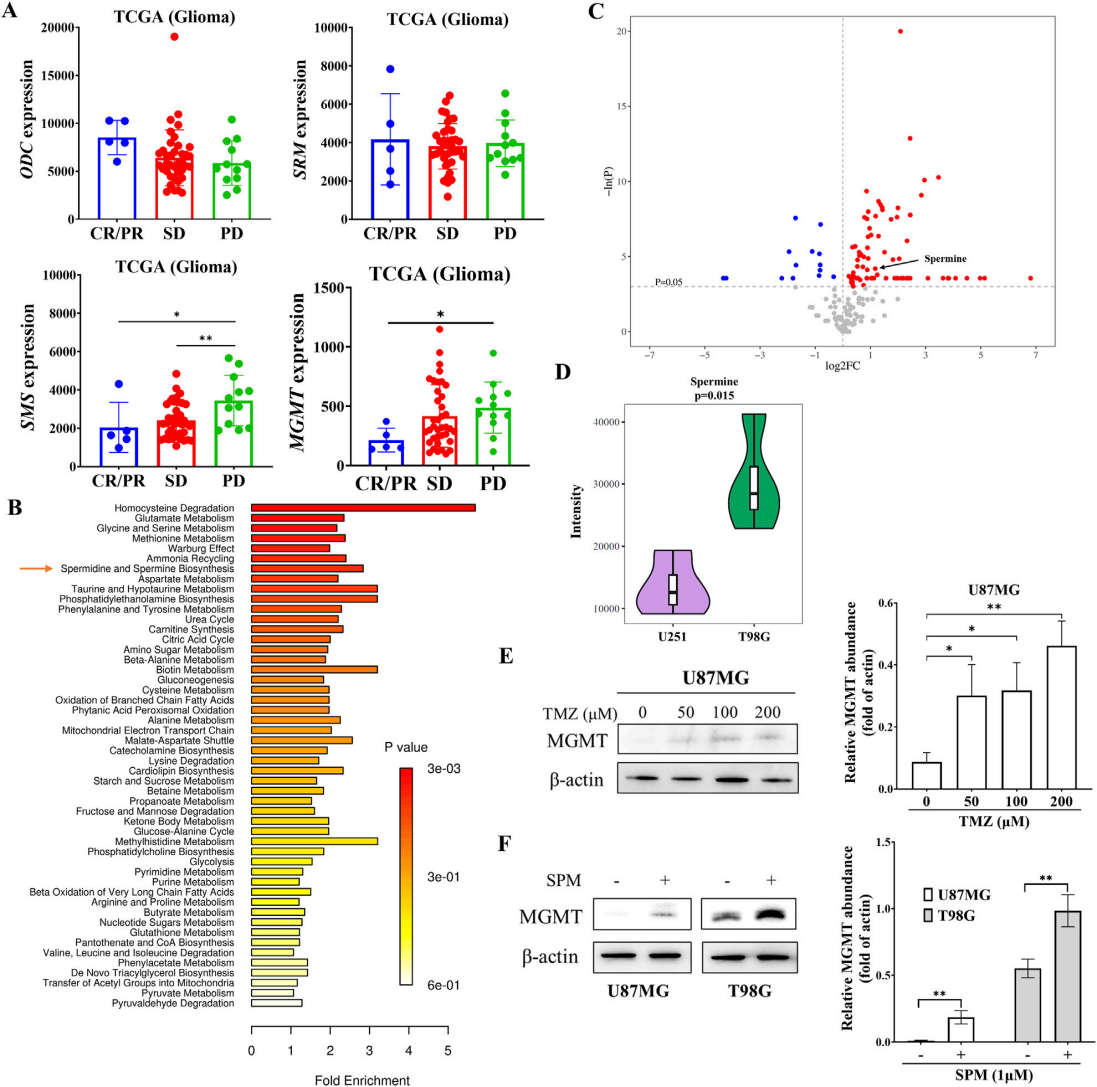
Elevated expression of SMS and increased spermine levels in TMZ-resistant glioma. **(A)** mRNA expression levels of ODC, SRM, and SMS in glioma patients treated with temozolomide. Data were obtained from the TCGA database. CR: complete response; PR: partial response; SD: stable disease; PD: progressive disease. Student’s t-test, *p < 0.05; **p < 0.01. **(B)** Metabolite enrichment analysis in T98G versus U251 cells. **(C)** Pathway enrichment analysis based on metabolite sets comparing U251 and T98G cells. **(D)** Violin plot showing spermine levels in U251 and T98G cells derived from univariate statistical analysis. **(E)** MGMT protein levels in U87MG cells treated with the indicated concentrations of TMZ for 24 h. Student’s t-test, *p < 0.05; **p < 0.01. **(F)** MGMT protein levels in U87MG and T98G cells treated with 1 μM spermine for 24 h. Student’s t-test, **p < 0.01.

High levels of polyamines have been associated with the progression of various cancers, including those of the breast, colon, and prostate. However, the relationship between polyamines and glioma remains unclear. Analysis of TCGA data from glioma patients treated with TMZ (Figure 1A) revealed that the expression of spermine synthase (SMS) was significantly higher in patients with progressive disease (PD) compared to those with a complete response (CR), partial response (PR), or stable disease (SD). In contrast, the expressions of ODC and SRM, which encode two other key enzymes in polyamine biosynthesis, showed no significant changes following TMZ treatment. Consistent with previously reported data, MGMT expression was higher in patients with PD than in those with SD or CR/PR.

To address this observation, we performed a metabolomic analysis of both TMZ-sensitive and TMZ-resistant glioma cells (Ji et al., 2021). The metabolomic data revealed differences in spermidine and spermine biosynthesis between TMZ-sensitive U251 cells and TMZ-resistant T98G cells (Figures 1B,C). Spermine levels were significantly higher in T98G cells than in U251 cells (Figure 1D). Further metabolomic analysis of parental U251 cells and their acquired TMZ-resistant counterparts, U251/T cells, also showed that spermine accumulated in the TMZ-resistant cells (Supplementary Figure S2).

Additionally, the expression of MGMT in U87MG cells was increased in a dose-dependent manner after 24 h of TMZ treatment (Figure 1E). Consistent with this finding, spermine also increased MGMT expression in both U87MG and T98G cells (Figure 1F).

### 3.2 Spermine promoted the proliferation of TMZ-resistant glioma cells

**FIGURE 2:**
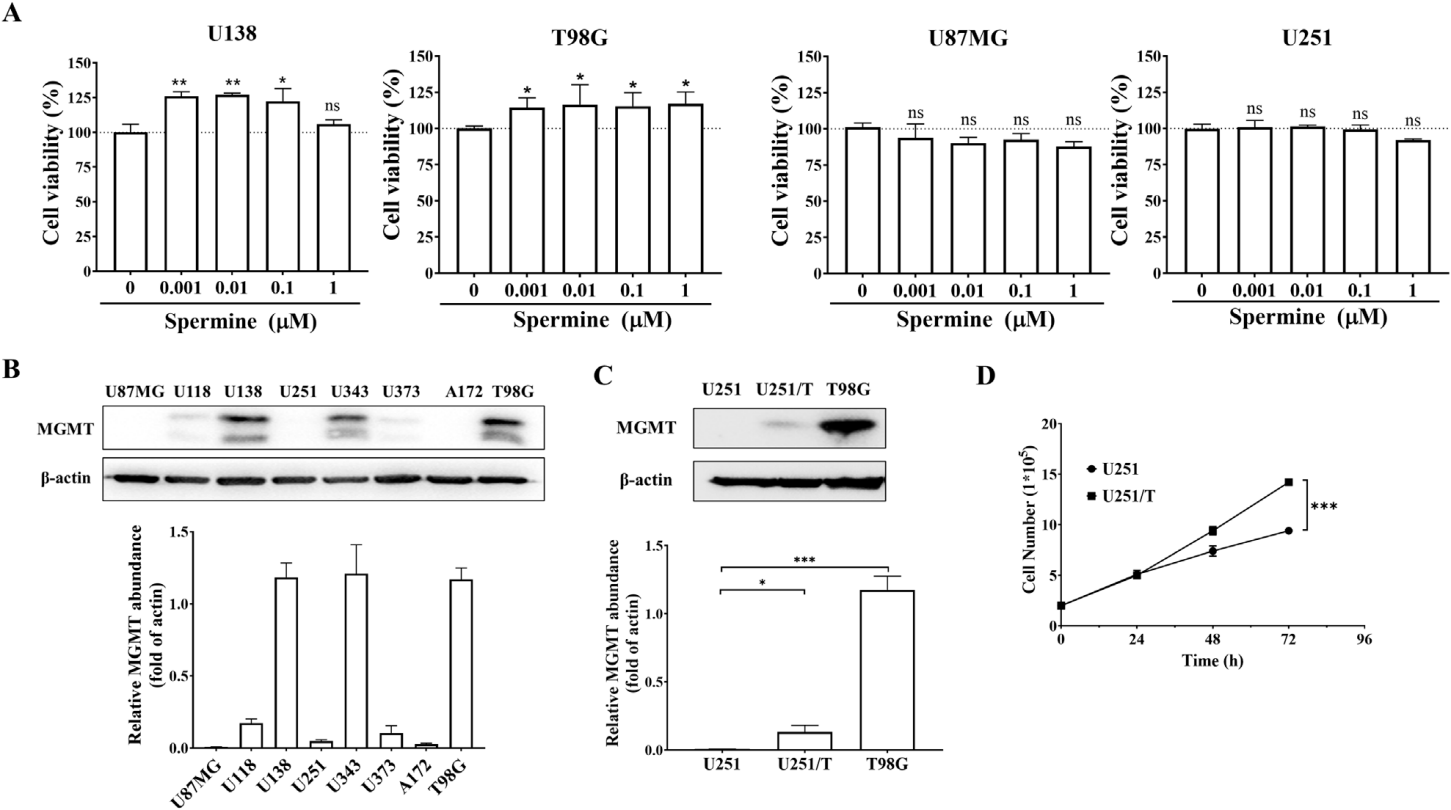
Spermine promotes proliferation in TMZ-resistant glioma cells. **(A)** Cell proliferation following treatment with the indicated concentrations of spermine for 72 h. Data are presented as the mean ± SD (n = 3). Student’s t-test, *p < 0.05, **p < 0.01, ns (not significant) p > 0.05, compared to the control group. **(B,C)** MGMT protein expression levels in different glioma cell lines. Student’s t-test, *p < 0.05; ***p < 0.001. **(D)** Growth curves of U251 and U251/T cells. Student’s t-test, ***p < 0.001.

Polyamines have been reported to play a key role in cancer cell proliferation. We investigated whether spermine promotes the proliferation of glioma cells. As shown in Figure 2A, treatment with spermine increased the proliferation of T98G and U138MG cells, which express high levels of MGMT and are resistant to TMZ (Figure 2B). In contrast, spermine did not promote the proliferation of U87MG and U251 cells, which are sensitive to TMZ. Additionally, we compared the proliferation rates of parental U251 cells and their acquired TMZ-resistant subtype, U251/T cells, which express MGMT (Figure 2C). U251/T cells grew significantly faster than the parental U251 cells (Figure 2D).

### 3.3 Pralsetinib inhibited the cell proliferation of TMZ-resistant glioma cells and induced cell apoptosis

**FIGURE 3:**
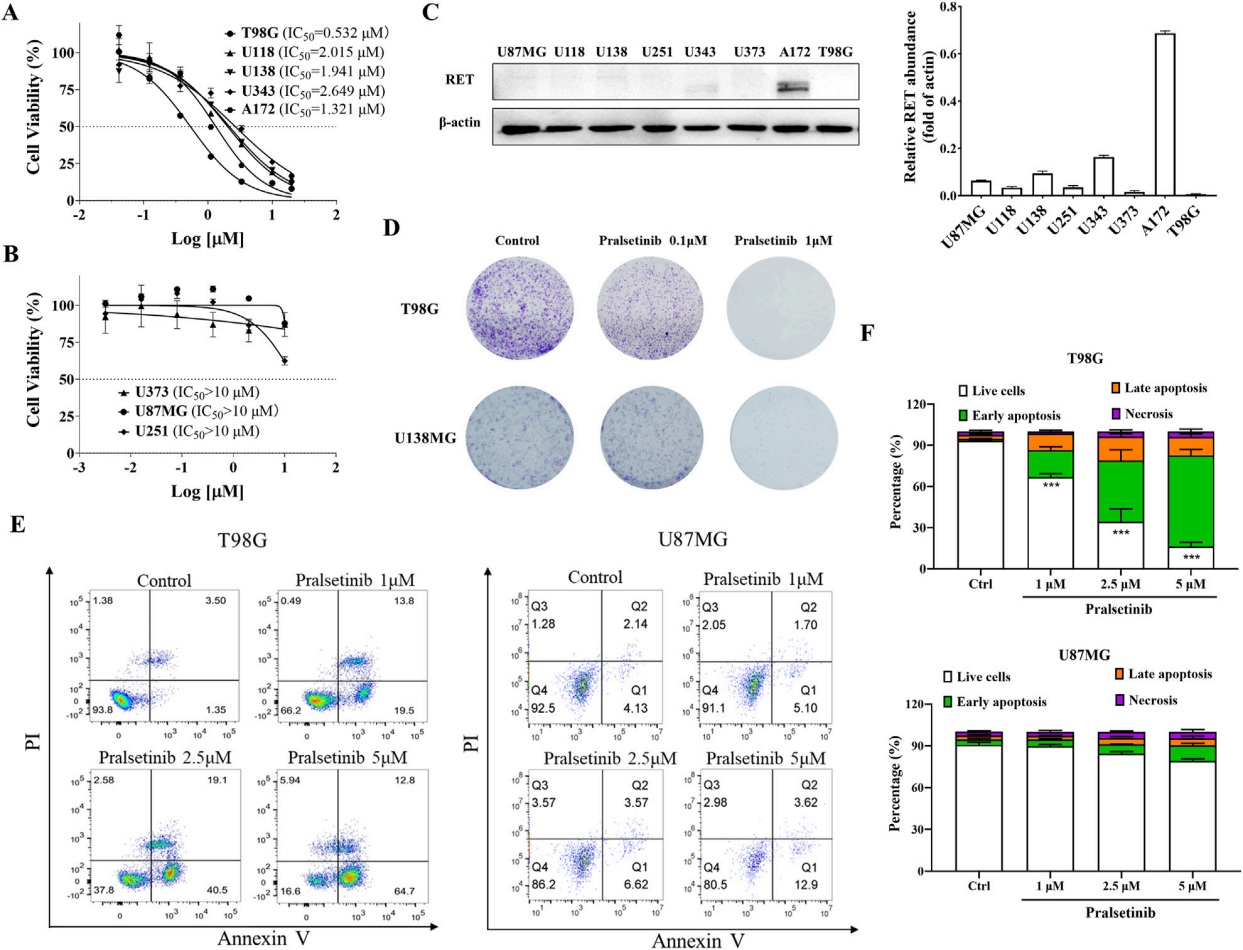
Pralsetinib inhibits cell proliferation and induces apoptosis in TMZ-resistant glioma cells. **(A)** Inhibitory effects of pralsetinib on cell proliferation in TMZ-resistant or RET-highly expressing glioma cell lines. **(B)** Effects of pralsetinib on proliferation in TMZ-sensitive glioma cells. **(C)** RET protein expression levels in various glioma cell lines. **(D)** Colony formation assay of T98G and U138MG cells treated with pralsetinib. **(E,F)** Apoptosis analysis of T98G and U87MG cells following 24-h treatment with pralsetinib. Student’s t-test, ***p < 0.001 versus control.

Since spermine selectively promoted the proliferation of TMZ-resistant glioma cells, we hypothesized that certain compounds might inhibit the proliferation of these resistant cells by blocking spermine biosynthesis or its downstream pathways. First, we screened a series of compounds in both TMZ-sensitive and TMZ-resistant glioma cells, while also considering their brain penetration capacity (Table 1; Supplementary Figure S3). Among these, pralsetinib—a highly selective RET inhibitor with good brain penetration—demonstrated strong anti-proliferative activity against T98G cells and against other TMZ-resistant glioma cells that do not express RET, and against the TMZ-sensitive A172 cell line, which exhibits high RET expression (Figures 3A–C). In contrast, pralsetinib showed weaker cytotoxicity in TMZ-sensitive cells such as U251, U87MG, and U373. Colony formation assays using T98G and U138MG cells further confirmed that pralsetinib strongly inhibits the proliferation of TMZ-resistant glioma cells (Figure 3D).

**TABLE 1:** The cytotoxicity of RTK inhibitors in glioma cells and their brain penetration.

Compound	Mean IC_50_ (μM)		Brain penetration
	T98G	U87MG	Pe (×10^−6^ cm/s)
Afatinib	4.96	>10	109.1
Anlotinib	1.18	>10	260.4
Apatinib	>10	>10	468.3
Axitinib	3.34	>10	181.8
Cabozantinib	2.21	2.40	173.4
Ceritinib	>10	6.95	590.5
Erlotinib	>10	>10	102.7
Gefitinib	>10	>10	458.6
Nilotinib	3.63	2.24	612.8
Osimertinib	>10	>10	693.7
Pralsetinib	0.532	>10	450.8
Sunitinib	4.37	>10	147.4
Zanubrutinib	>10	>10	88.9
Fluphenazine	ND	ND	688.8

ND: not detected.

We further investigated the effects of pralsetinib on the cell cycle and apoptosis in glioma cells. Pralsetinib did not alter the cell cycle distribution in any of the tested glioma cell lines at concentrations up to 5 μM (data not shown). However, it dose-dependently induced apoptosis in T98G cells (Figures 3E,F). In contrast, no significant apoptosis was observed in U87MG cells after treatment with pralsetinib at the same concentrations.

### 3.4 Pralsetinib blocked multiple signaling pathways activated by spermine

**FIGURE 4:**
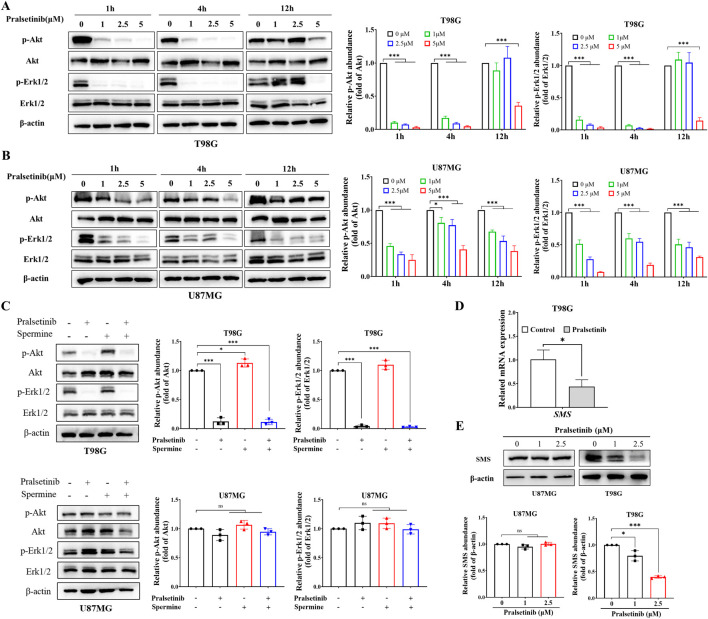
Pralsetinib inhibits multiple signaling pathways activated by spermine. **(A,B)** Phosphorylation levels of AKT and ERK1/2 in T98G and U87MG cells after treatment with pralsetinib for the indicated durations. Student’s t-test, *p < 0.05; ***p < 0.001. **(C)** Pralsetinib suppresses spermine-induced activation of the PI3K/AKT pathway in T98G cells at 24 h. Student’s t-test, *p < 0.05; ***p < 0.001, ns (not significant) p > 0.05. **(D)** SMS mRNA expression in T98G cells following 24-h treatment with pralsetinib. Student’s t-test, *p < 0.05. **(E)** SMS protein expression in T98G cells after 24-h exposure to pralsetinib. Student’s t-test, *p < 0.05; ***p < 0.001; ns p > 0.05.

As previously reported, pralsetinib inhibits multiple downstream signaling pathways. To investigate its effects in glioma cells, we examined the influence of pralsetinib on several key signaling cascades. As shown in Figure 4A, pralsetinib strongly inhibited the PI3K/AKT and ERK/MAPK pathways in T98G cells in a dose-dependent manner. These inhibitory effects occurred rapidly, within 1–4 h of treatment, and then gradually recovered. Consistent with the limited anti-proliferative effect of pralsetinib in TMZ-sensitive cell lines, only mild inhibition of the PI3K/AKT and ERK/MAPK pathways was observed in U87MG cells (Figure 4B). Furthermore, spermine treatment activated the PI3K/AKT pathway in T98G cells at 24 h, and this activation was effectively suppressed by pralsetinib (Figure 4C). In contrast, no such antagonistic effect of pralsetinib was observed in U87MG cells.

Interestingly, PI3K signaling is not only the downstream of polyamine but also regulates polyamine production (Rajeeve et al., 2013). Since pralsetinib suppresses the PI3K/AKT pathway, we hypothesized that it might also affect spermine levels in TMZ-resistant glioma cells. As shown in Figures 4D,E, both the mRNA and protein expression levels of SMS—a key enzyme in spermine synthesis—were downregulated in T98G cells after 12 h of pralsetinib treatment. In contrast, pralsetinib had a minimal effect on SMS protein expression in U87MG cells.

### 3.5 Pralsetinib suppressed tumor growth in TMZ-resistant glioma cells in xenograft mouse models

**FIGURE 5:**
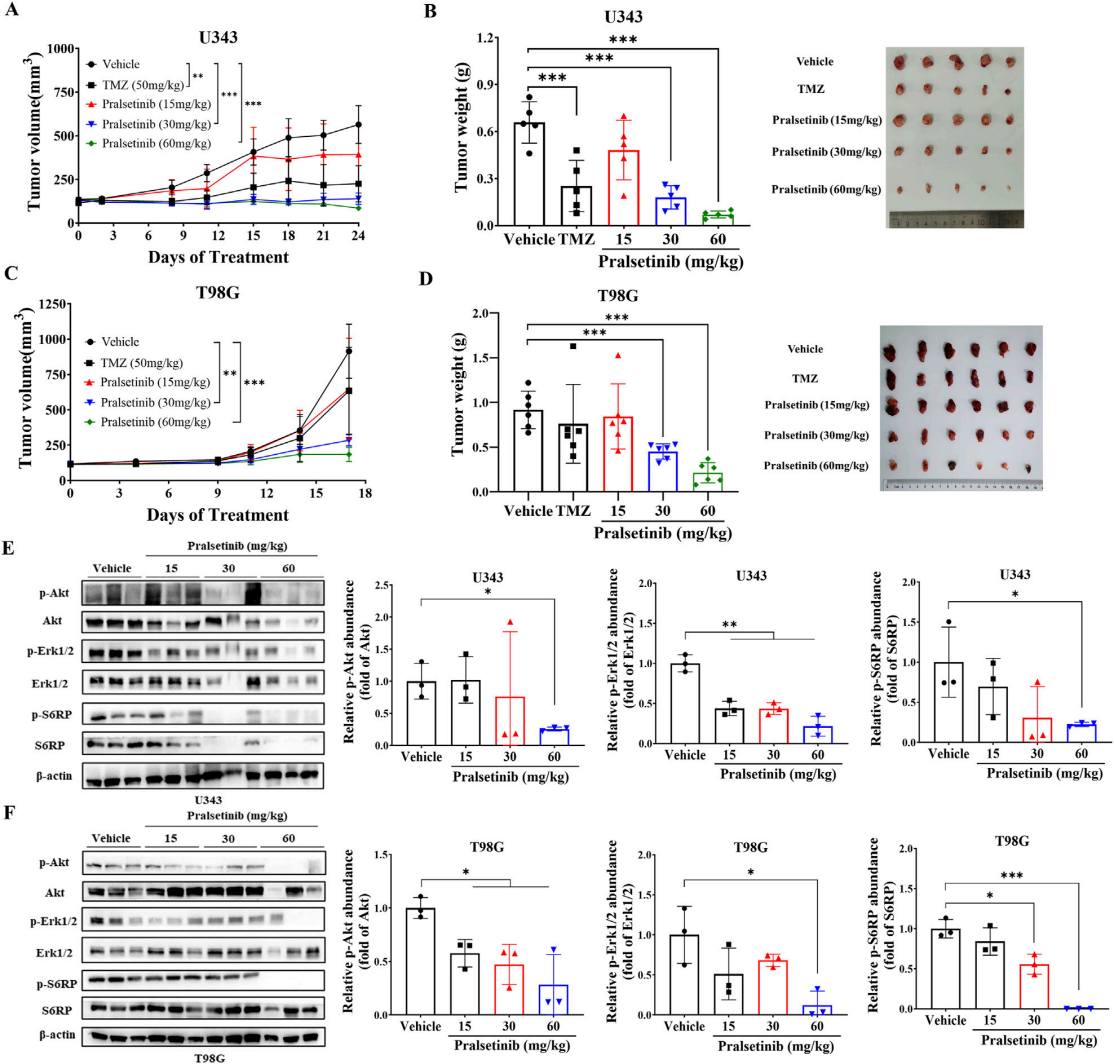
Pralsetinib suppresses tumor growth in xenograft mouse models of TMZ-resistant glioma. **(A)** Tumor growth curves in U343 xenograft mice treated with pralsetinib. Student’s t-test, **p < 0.01, ***p < 0.001 versus vehicle group on day 24; n = 5. **(B)** Tumor weight in the U343 xenograft model. Student’s t-test, ***p < 0.001 versus vehicle group; n = 6. **(C)** Tumor growth curves in T98G xenograft mice treated with pralsetinib. Student’s t-test, **p < 0.01; ***p < 0.001 versus vehicle group on day 17. **(D)** Tumor weight in the T98G xenograft model. Student’s t-test, ***p < 0.001 versus vehicle group. **(E,F)** Pralsetinib inhibits phosphorylation of AKT and ERK1/2 in tumor tissues; n = 3. Student’s t-test, *p < 0.05; ***p < 0.001.

Since pralsetinib robustly inhibited the proliferation of TMZ-resistant glioma cells *in vitro*, we next evaluated its antitumor efficacy *in vivo*. In a subcutaneous xenograft mouse model established with human U343 glioma cells (Figures 5A,B), oral administration of pralsetinib dose-dependently suppressed tumor growth. At a dose of 60 mg/kg per day, pronounced tumor shrinkage was observed, resulting in 89.5% tumor growth inhibition (TGI), compared to 61.9% TGI in the TMZ-treated group. In a separate T98G subcutaneous xenograft model that was resistant to TMZ treatment (Figures 5C,D), pralsetinib also exhibited significant antitumor activity. At a high dose of 60 mg/kg daily, TGI reached 76.6%, compared to 17.2% in the TMZ group. Furthermore, in pralsetinib-treated mice, phosphorylated levels of Erk1/2, AKT, and downstream S6RP were reduced in tumor tissues from both models (Figures 5E–F).

**FIGURE 6:**
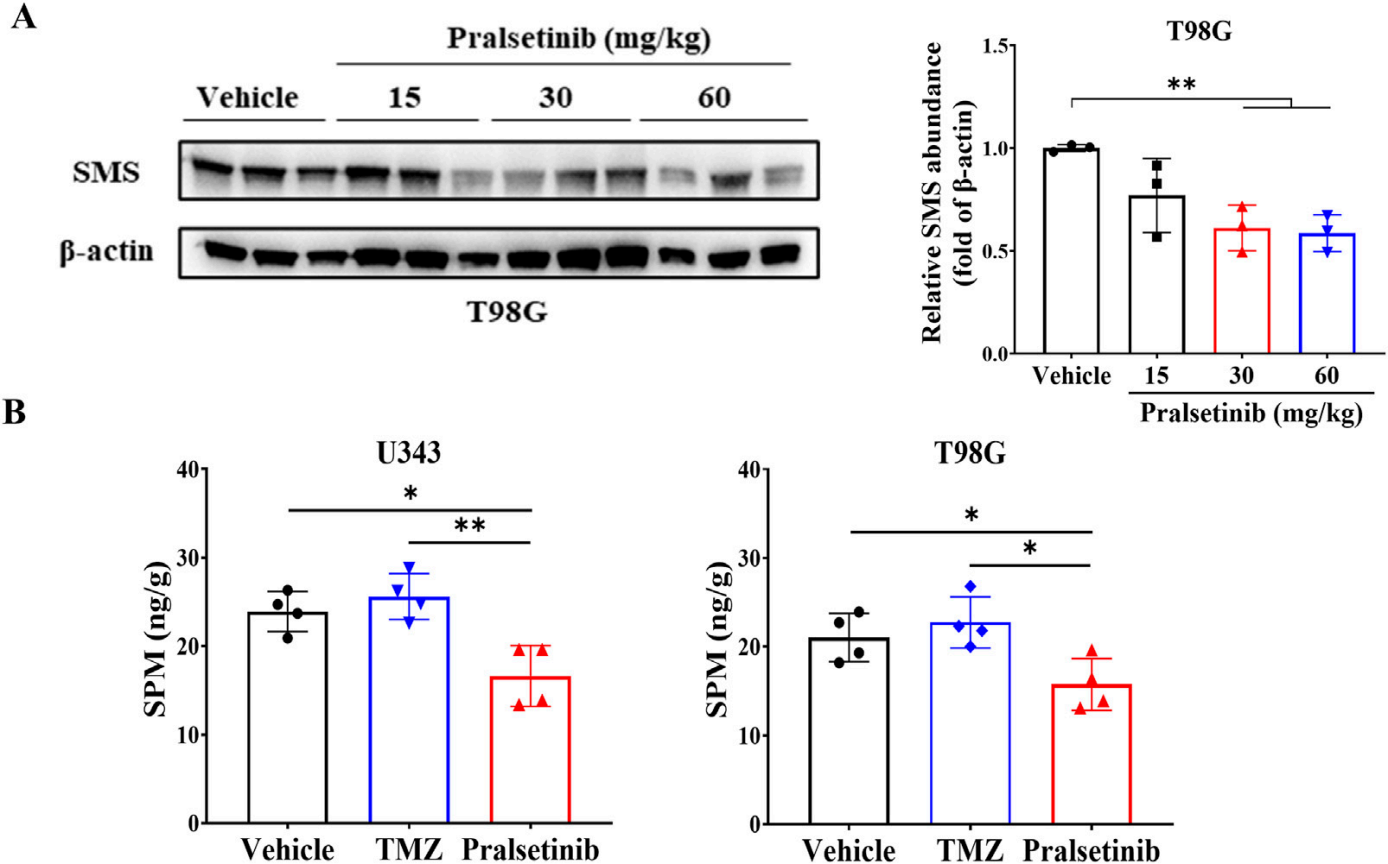
Pralsetinib downregulates SMS protein expression and reduces spermine levels in tumor tissues. **(A)** SMS protein levels were decreased following pralsetinib treatment; n = 3. Student’s t-test, **p < 0.01. **(B)** Spermine concentrations in tumor tissues are reduced after pralsetinib treatment at doses of 30 mg/kg in the U343 model and 60 mg/kg in the T98G model; n = 4. Student’s t-test, *p < 0.05, **p < 0.01.

Furthermore, we examined the expression of SMS in tumor tissues derived from T98G xenografts. As expected, SMS protein levels were downregulated following pralsetinib treatment (Figure 6A). Consistent with this finding, LC-MS/MS analysis revealed a statistically significant reduction in spermine levels in tumor tissues from pralsetinib-treated groups compared to the vehicle group in both the U343 and T98G mouse models (Figure 6B). In contrast, putrescine and spermidine levels remained unchanged in both models (Supplementary Figure S4).

## 4 Discussion

Glioma is one of the most malignant tumors in adults, particularly high-grade glioma. As with other solid tumors, altered cellular metabolism is a well-established hallmark of glioma (Bi et al., 2020; Des et al., 2021). Numerous studies have demonstrated that metabolic dysregulation, including in glycolysis, glutamine, fatty acid, cholesterol metabolism, and lactic acidosis, plays a crucial role in glioma development, invasion, angiogenesis, and resistance to chemotherapy and radiation (Kant et al., 2020; Tardito et al., 2015; Kathagen-Buhmann et al., 2016; Duan et al., 2018; Taib et al., 2019). Alterations in polyamine metabolism have also been identified in various cancers, such as breast, prostate, gastric, and brain tumors, including neuroblastoma and glioma (Ernestus et al., 2001; Ernestus et al., 1996). In our study, we found that SMS, a key enzyme in spermine production, was highly expressed in glioma patients who responded poorly to TMZ treatment. Metabolomic analysis further revealed elevated spermine levels in TMZ-resistant glioma cells. Functionally, spermine promoted the proliferation of TMZ-resistant glioma cells and was found to increase MGMT expression. These findings suggest that spermine may contribute to the development of TMZ resistance in glioma.

TMZ is the first-line treatment for glioma; however, resistance frequently develops following TMZ-based therapy. The mechanisms underlying TMZ resistance are multifactorial and include MGMT overexpression, activation of glioma stem cells, and metabolic dysregulation. New therapeutic strategies, particularly tyrosine kinase inhibitors (TKIs), have been investigated in clinical trials to overcome TMZ resistance. Unfortunately, most have failed due to poor blood–brain barrier penetration and the immunosuppressive glioma microenvironment. In this study, we found that pralsetinib, a selective RET inhibitor approved for the treatment of RET fusion–positive non-small cell lung cancer and thyroid cancer, exhibits potent anti-tumor activity against TMZ-resistant glioma cells both *in vitro* and *in vivo*. Moreover, we observed that pralsetinib reduced spermine levels in resistant glioma cells. These results suggest that targeting polyamine metabolism may represent a novel therapeutic strategy for brain tumors, particularly glioma. Supporting this concept, DFMO, an ornithine decarboxylase (ODC) inhibitor, was recently approved for the treatment of neuroblastoma (Tangella et al., 2023; Cecile et al., 2023). Additionally, other compounds targeting key enzymes in polyamine metabolism, such as S-adenosylmethionine decarboxylase (AdoMetDC), spermidine synthase (SRM), and spermine synthase (SMS), are currently in preclinical or clinical development (Casero et al., 2018).

The pathogenesis of glioma often involves dysregulated signaling in key oncogenic pathways, such as constitutive PI3K/AKT activation, EGFR amplification/overexpression, and other aberrant growth factor signals (Woo et al., 2020; Zhao et al., 2021; The Cancer Genome Atlas Research Network, (2008)). These signaling networks play a central role in the metabolic reprogramming characteristic of glioma (Des et al., 2021). Previous studies have indicated that both the PI3K/AKT and RAS/MAPK pathways are closely linked to polyamine metabolism (Des et al., 2021). It has been reported that PI3K/AKT and RAS/MAPK pathways are associated with polyamine metabolism (Roy et al., 2008; Gomes et al., 2017; Zabala-Letona et al., 2017; Wang et al., 2017). Moreover, spermine has been shown to activate the PI3K/AKT pathway through a positive feedback mechanism. Our data demonstrate that pralsetinib not only inhibits the spermine-induced activation of the PI3K/AKT pathway but also downregulates SMS expression in TMZ-resistant glioma cells. These findings provide new insights into the pleiotropic mechanisms of tyrosine kinase inhibitors (TKIs). Other TKIs, such as osimertinib and crizotinib, have also been reported to modulate metabolic processes in tumor cells beyond their canonical inhibition of oncogenic kinases (Ye et al., 2021; Eltayeb et al., 2024). These agents are currently under clinical evaluation in patients with glioma and brain-metastatic lung cancer (Chen et al., 2021; Zhao et al., 2022; Das et al., 2015).

In conclusion, our study uncovered a novel pharmacological activity of pralsetinib against TMZ-resistant glioma, suggesting its potential as a therapeutic candidate for expanding the clinical indications of pralsetinib.

## Data Availability

The original contributions presented in the study are included in the article/Supplementary Material, further inquiries can be directed to the corresponding authors.

## Supplementary material

SUPPLEMENTARY FIGURE S1 Schematic diagram of the μFlux apparatus.

SUPPLEMENTARY FIGURE S2 Metabolomic analysis of U251 and U251/T cells. **(A)** Metabolite enrichment analysis in U251 versus U251/T cells. **(B)** Violin plot illustrating spermine levels in U251 and U251/T cells based on univariate statistical analysis.

SUPPLEMENTARY FIGURE S3 Cytotoxicity profiles of TKIs in U87MG and T98G glioma cells. **(A,B)** Cell inhibition curves following treatment with various concentrations of TKIs for 72 h **(C)** IC_50_ values of the tested TKIs.

SUPPLEMENTARY FIGURE S4 Levels of putrescine (PUT) and spermidine (SPD) in tumor tissues. **(A)** PUT and SPD concentrations in T98G xenograft tumors after treatment with pralsetinib (60 mg/kg). **(B)** PUT and SPD concentrations in U373 xenograft tumors after treatment with pralsetinib (30 mg/kg).
