# Supplementary figures and images for: The RET inhibitor pralsetinib suppresses TMZ-resistant glioma growth by regulating spermine production
Source: Front Pharmacol. 2025 Oct 31;16:1671798. doi: 10.3389/fphar.2025.1671798 (PMC12615384; doi:10.3389/fphar.2025.1671798)

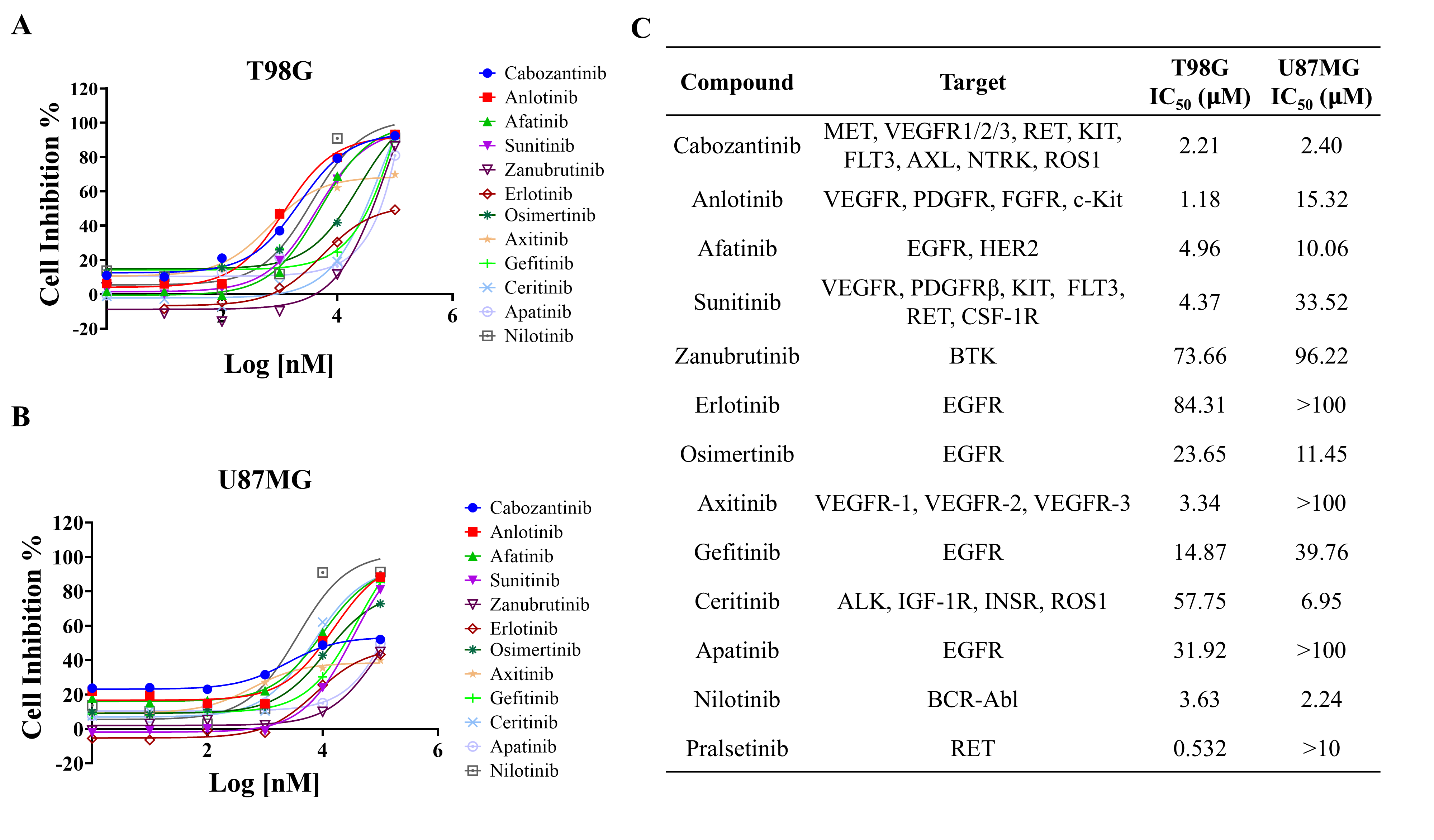

Supplement: Supplementary file 1 [file Image3.tif]

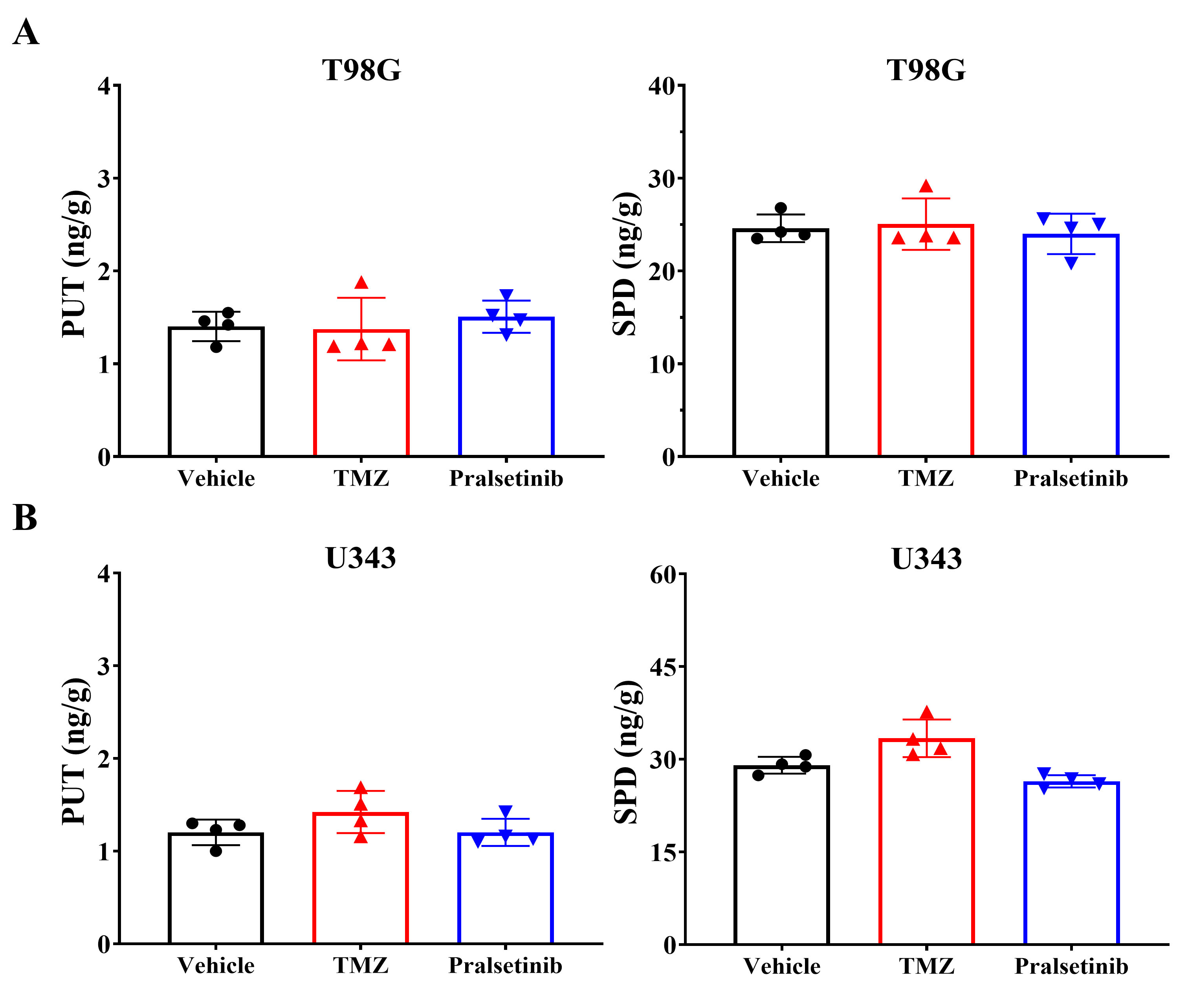

Supplement: Supplementary file 2 [file Image4.tif]

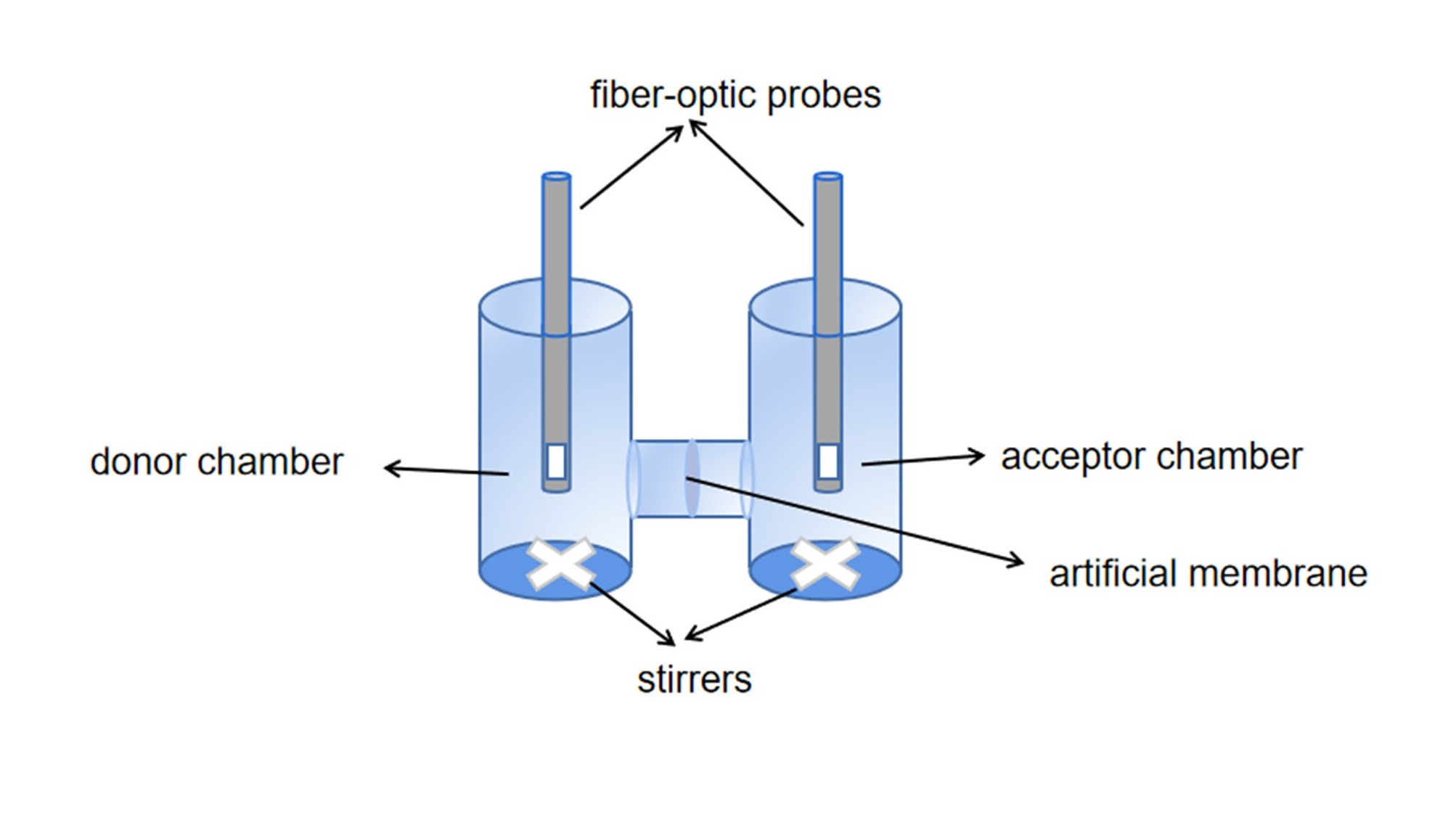

Supplement: Supplementary file 3 [file Image1.jpeg]

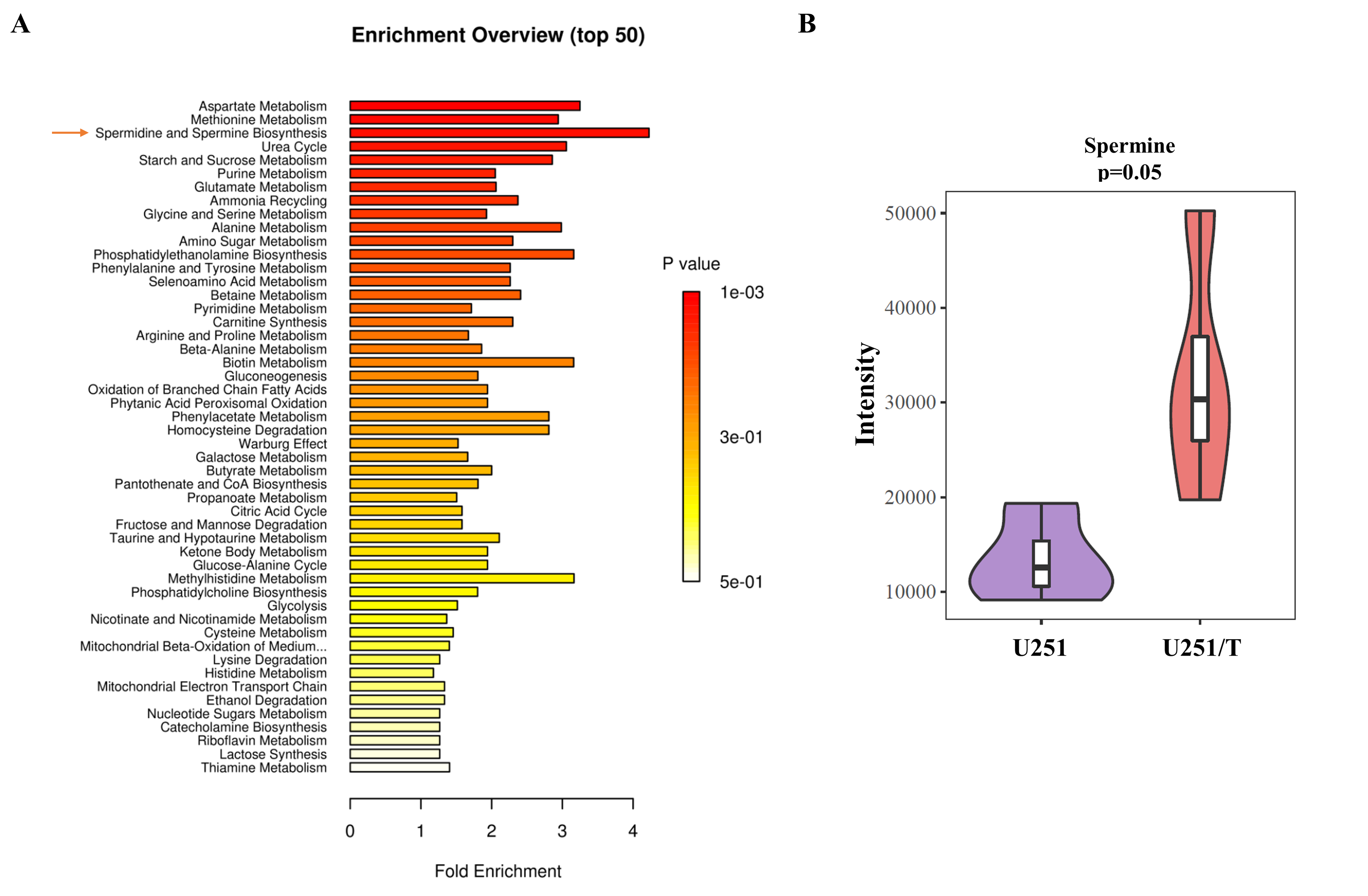

Supplement: Supplementary file 4 [file Image2.tif]
